# Genome wide SNP identification in chickpea for use in development of a high density genetic map and improvement of chickpea reference genome assembly

**DOI:** 10.1186/1471-2164-15-708

**Published:** 2014-08-23

**Authors:** Amit A Deokar, Larissa Ramsay, Andrew G Sharpe, Marwan Diapari, Anoop Sindhu, Kirstin Bett, Thomas D Warkentin, Bunyamin Tar’an

**Affiliations:** Crop Development Centre, Department of Plant Sciences, University of Saskatchewan, 51 Campus Dr, Saskatoon, SK S7N 5A8 Canada; National Research Council Canada, 110 Gymnasium Place, Saskatoon, SK S7N 0 W9 Canada

**Keywords:** *Cicer arietinum*, Genotyping-by-sequencing, Restriction site associated DNA (RAD) markers, Illumina GoldenGate and Genetic mapping

## Abstract

**Background:**

In the whole genome sequencing, genetic map provides an essential framework for accurate and efficient genome assembly and validation. The main objectives of this study were to develop a high-density genetic map using RAD-Seq (Restriction-site Associated DNA Sequencing) genotyping-by-sequencing (RAD-Seq GBS) and Illumina GoldenGate assays, and to examine the alignment of the current map with the kabuli chickpea genome assembly.

**Results:**

Genic single nucleotide polymorphisms (SNPs) totaling 51,632 SNPs were identified by 454 transcriptome sequencing of *Cicer arietinum* and *Cicer reticulatum* genotypes. Subsequently, an Illumina GoldenGate assay for 1,536 SNPs was developed. A total of 1,519 SNPs were successfully assayed across 92 recombinant inbred lines (RILs), of which 761 SNPs were polymorphic between the two parents. In addition, the next generation sequencing (NGS)-based GBS was applied to the same population generating 29,464 high quality SNPs. These SNPs were clustered into 626 recombination bins based on common segregation patterns. Data from the two approaches were used for the construction of a genetic map using a population derived from an intraspecific cross. The map consisted of 1,336 SNPs including 604 RAD recombination bins and 732 SNPs from Illumina GoldenGate assay. The map covered 653 cM of the chickpea genome with an average distance between adjacent markers of 0.5 cM. To date, this is the most extensive genetic map of chickpea using an intraspecific population. The alignment of the map with the CDC Frontier genome assembly revealed an overall conserved marker order; however, a few local inconsistencies within the *Cicer arietinum* pseudochromosome 1 (Ca1), Ca5 and Ca8 were detected. The map enabled the alignment of 215 unplaced scaffolds from the CDC Frontier draft genome assembly. The alignment also revealed varying degrees of recombination rates and hotspots across the chickpea genome.

**Conclusions:**

A high-density genetic map using RAD-Seq GBS and Illumina GoldenGate assay was developed and aligned with the existing kabuli chickpea draft genome sequence. The analysis revealed an overall conserved marker order, although some localized inversions between draft genome assembly and the genetic map were detected. The current analysis provides an insight of the recombination rates and hotspots across the chickpea genome.

**Electronic supplementary material:**

The online version of this article (doi:10.1186/1471-2164-15-708) contains supplementary material, which is available to authorized users.

## Background

Chickpea (*Cicer arietinum* L., 2n = 16) is the second most widely grown food legume crops after common bean, with annual production of 11.6 M tons [1]. Chickpea grains are a good source of many essential mineral nutrients, protein, and dietary fiber and are low in saturated fat (http://ndb.nal.usda.gov/ndb/foods/show/4778). The chickpea crop helps to restore and maintain soil fertility through symbiotic nitrogen fixation. Globally more than 90% of chickpea production occurs in the semi-arid tropics of Asia and Africa [1]; however during the last three decades acreage in non-traditional areas such as Australia, Canada and USA has increased rapidly. In the traditional production regions, chickpea is considered as a low input crop and is mainly grown on residual soil moisture. In these areas terminal drought, fusarium wilt and pod borer are some of the major constraints to chickpea production; whereas, in non-traditional, temperate growing areas ascochyta blight, low temperatures and end of season frost are the major constraints [2–4]. In spite of these constraints, considerable progress has been made in chickpea improvement using conventional breeding approaches. Several cultivars with improved resistance to different biotic and abiotic stresses have been commercialized. However, chickpea productivity globally is still very low (0.8 t/ha) [1] and has remained stagnant in the last two decades [5]. In contrast, application of modern genomic approaches has contributed significantly to the overall yield improvement in many cereal crops [6, 7].

Genetic maps may serve as the basis for genetic studies of various agronomic traits through mapping of major genes and QTLs. They are also of practical benefit in the application of genomics through fine mapping, map-based cloning and development of tightly linked markers for marker-assisted selection (MAS). Limited genomic resources and low levels of genetic variability in the primary gene pool, however, have restricted the practical application of genetic mapping in chickpea [8]. During the last two decades several genetic maps have been developed for chickpea using restriction fragment length polymorphism (RFLP), amplified fragment length polymorphism (AFLP), cleaved amplified polymorphic sequence (CAPS), simple sequence repeat (SSR), and single nucleotide polymorphism (SNP) markers based on the mapping of populations derived from intra- and interspecific crosses [9–12]. In addition, several genomic resources including large collections of expressed sequence tags (ESTs), SSRs markers and several thousands of SNPs have been developed in chickpea in recent years [13]. The availability of these genetic and genomic resources will facilitate in depth genetic study and in turn will aid in the development of chickpea cultivars with improved resistance to biotic and abiotic stresses and desirable agronomic traits.

Next generation sequencing (NGS) technology has become powerful tool for detecting large numbers of SNPs in a relatively short time frame [14]. SNPs are the most abundant class of markers present in both plant and animal genomes [15]. The frequency of SNPs in plants varies from one SNP per 16 bp in *Eucalyptus* species [16] to one SNP per 7000 bp in tomato [17]. In chickpea, SNP frequencies of one per 36 bp [18] to one per 973 bp [19] have been observed. However, the frequency calculations are highly influenced by the diversity and number of accessions used in the analysis. The high frequency of SNPs in the chickpea genome compared to SSR markers (one SSR in every 4.85 kb) [20] makes SNPs an ideal marker system for development of high density genetic map and has now become the marker of choice among chickpea researchers [11, 21].

In parallel to the development of sequencing technologies, several new technologies for large scale SNP genotyping have been developed. These technologies can integrate up to one million SNPs and several folds of multiplexing per assay. Among these, Illumina GoldenGate and Infinium genotyping platforms have been widely used in many crops including soybean [22], wheat [23], maize [24], rice [25], sunflower [26] and lentil [27]. These genotyping platforms have been used to generate high density genetic linkage maps with the average distance of the adjacent markers of less than 1 cM in soybean [28], apple [29] and tomato [30].

The efficiency of genome-wide marker-trait association mostly depends on SNP marker density and distribution. Therefore, it is important to develop SNP based genotyping platforms that allow association study to be conducted in chickpea. Cost involved in the development of an array-based genotyping platform mostly depends on initial SNP discovery, SNP selection and development of array platforms.

Recent advances in DNA sequencing technologies have generated several cost-effective SNP discovery and genotyping platform such as genotyping-by-sequencing (GBS) [31], complexity reduction of polymorphic sequences (CRoPS) [32], and restriction site associated DNA (RAD) [33]. RAD markers together with NGS have provided an efficient method that can simultaneously detect thousands of SNPs and provide genotypic data of several hundred samples with no prior genome sequence information. The effectiveness of RAD markers for development of high density genetic map and QTL analysis [34], and association mapping [35] has been successfully demonstrated in several plant species.

Whole genome re-sequencing of more than 90 chickpea cultivars of desi type (smaller seeds of angular shape with dark seed coat), kabuli type (large owl's or ram's head shaped seeds with cream-colored seed coat) and wild accessions has been completed recently [36, 37]. A high density genetic map is a primary requisite to anchor the assembled scaffolds to chromosomes. The previous genetic linkage map [21, 38] only allowed 65.2% of the assembled scaffolds to be anchored on the final eight chromosomes of the kabuli chickpea [36]. Therefore, a dense genetic map with additional markers would be desirable to allow anchoring of a higher percentage of the assembled scaffolds.

In the early phase of chickpea genomic research, many linkage maps were developed using interspecific crosses between *Cicer arietinum* and *C. reticulatum* due to the low polymorphism among the cultivated chickpea genotypes. In the present study, we generated one of most comprehensive and high density chickpea genetic maps from intraspecific population available to date. We also demonstrated the potential use of this map as tool for improving the whole genome assembly. Comparison of this high density genetic map with the whole genome sequencing data revealed the recombination landscape in the current population. The identified SNP markers with anchored positions on the genetic and physical maps can be used for fine scale QTL mapping and candidate gene identification.

## Materials and methods

### Experimental design

**Figure 1:**
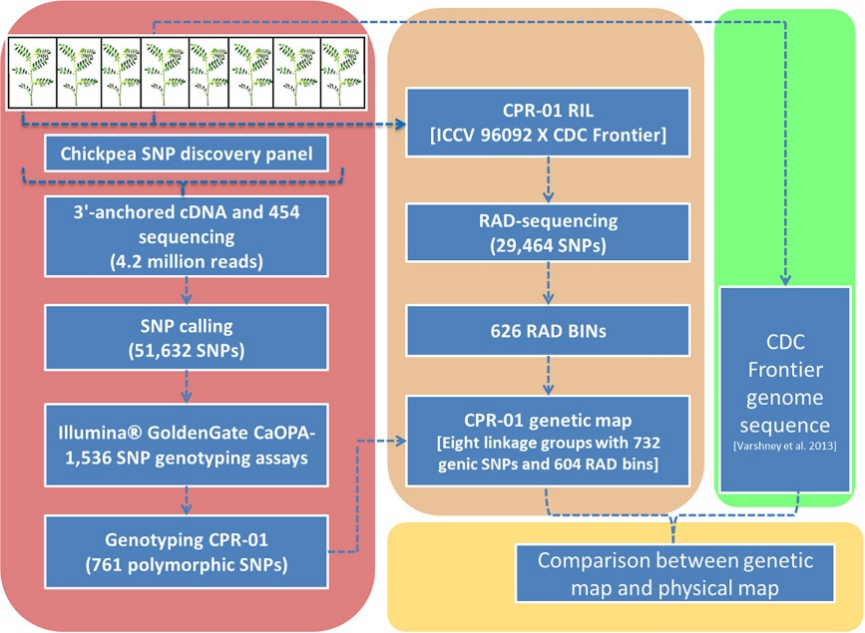
**Schematic diagram describing the experimental approach to develop a high density genetic map of chickpea.** A SNP panel of eight chickpea accessions representing desi, kabuli and wild species were used for SNP discovery. 3’-anchored cDNA was sequenced using the Roche 454 Titanium sequencing. SNP calling was done using in-house developed pipeline. Illumina 1536 SNP genotyping assays were developed. CPR-01 was genotyped using Illumina GoldenGate and Restriction site associated DNA (RAD) markers. An integrated high density genetic map was generated from the two data sets. A comparative study between CPR-01 and the recently released draft chickpea genome sequence (Varshney et al. 2013) was conducted. See main text for details.

The schematic outline of the experimental protocol is given in Figure 1. Large scale SNP discovery and genotyping were done using two high-throughput methods: First, transcriptome sequencing using 3’-anchored cDNA 454 sequencing and genotyping using Illumina GoldenGate assays, and second using genotyping-by-sequencing RAD-seq. A high density linkage map of CPR-01 was constructed using 1,336 SNP loci (604 RAD bins and 732 genic SNPs). The high-density CPR-01 map was compared with CDC Frontier genome sequences.

### Plant material

Eight chickpea genotypes: Amit, CDC Frontier, CDC Xena, ICC 12512–1, ICCV 96029, Y9563-28, Cr 5–10 and ILWC 118, were used for SNP discovery. These lines represent cultivated chickpea (*Cicer arietinum* L.), including desi and kabuli market classes, as well as wild species (*Cicer reticulatum*) accessions (Table 1).

**Table 1:** **Results of transcriptome sequencing and SNP discovery in cultivated and wild chickpea in comparison to the reference CDC Frontier genome** [36]

Chickpea accessions	Species (Desi/Kabuli)	Important traits	Total 454 reads	Number of SNPs	Average read depth
CDC Frontier	*Cicer arietinum* L. (Kabuli)	Yield, Ascochyta blight resistance, photoperiod sensitivity	490,245	NA	NA
Amit	*Cicer arietinum* L. (Kabuli)	Yield, Ascochyta blight resistance	496,109	1,592	7
CDC Xena	*Cicer arietinum* L. (Kabuli)	Yield, seed quality	531,970	1,813	7
ICC 12512-1	*Cicer arietinum* L. (Desi)	Ascochyta blight resistance	507,802	2,872	8
ICCV 96029	*Cicer arietinum* L (Desi)	Earliness, double podding, photoperiod insensitivity	520,733	3,286	8
Y9563-28	*Cicer arietinum* L (Desi)	Earliness, double podding	509,682	2,898	8
Cr 5-10	*Cicer reticulatum* L. (wild)	Rust (*Uromyces ciceris-arietini*) resistance	605,001	28,712	10
ILWC 118	*Cicer reticulatum* L. (wild)	Ascochyta blight resistance	560,322	28,071	11
Total			4,221,864	51,632^*^	

*Total non-redundant SNPs detected across all genotypes.

CPR-01, a bi-parental mapping population of 92 RILs derived from a cross between ICCV 96029 and CDC Frontier [9] was used to map the SNPs. CDC Frontier is a kabuli type chickpea cultivar released in 2003 by the Crop Development Centre, University of Saskatchewan and is the most widely grown kabuli cultivar in western Canada [39]. CDC Frontier has medium seed size, is day length sensitive, and moderately resistance to ascochyta blight. ICCV 96029 is a desi type cultivar released in 2000 by ICRISAT. It has a small seed size, is day length insensitive and highly susceptible to ascochyta blight.

### SNP discovery, SSR discovery and Illumina GoldenGate genotyping

Plant tissue for RNA extraction was collected from each genotype individually at various developmental stages, including seedling emergence, 8–10 node stage, early flowering stage, early pod stage and early senescence. Total RNA was extracted using the RNeasy Plant Mini Kit (Qiagen) and treated with DNase I (Invitrogen) to remove DNA contamination. Two micrograms of total RNA at each developmental stage were pooled. *Aci* I digested 3’-anchored cDNA libraries were constructed as previously described [40, 41]. Each line was sequenced using the Roche 454 Titanium sequencing protocol following the procedure described by Margulies et al. [42] and Titanium chemistry as described in the protocols supplied by the manufacturer (Roche, Laval, Quebec). The libraries were sequenced at the National Research Council Canada, Saskatoon, SK, Canada. Sequencing reads were aligned directly to the chickpea scaffold assembly V0.1 using GMAP [43] to produce SAM file format. SNP discovery for each genotype was undertaken using Samtools Version = 0.1.18 (http://samtools.sourceforge.net/). SNPs present in at least two of the eight accessions were filtered for further analysis. In order to design oligos for the Illumina GoldenGate array (Illumina Inc., San Diego, CA), sequences with a minimum of 60 bp flanking the SNP were selected. Further, SNPs were selected based on the Illumina Assay Design Tool (ADT) score (above 0.4 and preferentially above 0.6) and even distribution across the *Medicago* genome. This strategy was designed and implemented prior to the availability of the chickpea pseodochromosomes (Cicer*_*arietinum_GA_v1.0 pseudochromosomes). The same strategy has been successfully implemented in lentil SNP genotyping assay design [27]. Intron-exon boundaries within 60 bp of the SNP flanking regions (121 bp sequence) were predicted using BLASTN analysis with the *Medicago* genome and sequences located within a single exon were selected. Finally, 1,536 SNPs were chosen for Illumina GoldenGate assay for the production of an Oligo Pooled Array (Ca1536 GoldenGate OPA). Twenty SNPs were randomly selected for validation using allele-specific PCR assays (KASP™ Assays, LGC Genomics).

Gene ontology (GO) analysis was conducted on SNPs containing transcript sequences using Blast2GO program [44]. These transcripts were also annotated into Kyoto Encyclopedia of Genes and Genomes (KEGG) pathways with KEGG Automatic Annotation Server (KAAS), using *Arabidopsis thaliana* and *Oryza sativa* data sets [45]. A SnpEff v3.0 open source program was also used for variant annotation and effect prediction of SNPs (http://snpeff.sourceforge.net/) [46].

SSR identification was done using the QDD software program [47] with the following criteria: a minimum of eight repeats for dinucleotide motifs, six repeats for trinucleotide motifs and five repeats for tetranucleotide motifs and a minimum length of 100 bp for the PCR product.

SNP genotyping was performed using the Illumina GoldenGate platform, following the standard assay protocol (http://www.illumina.com/technology/goldengate_genotyping_assay.ilmn). Products generated by this assay were read with an Illumina HiScan (Illumina Inc., San Diego, CA) and the resulting data were clustered for allele calling using GenomeStudio software version 2010.3 (Illumina Inc., San Diego, CA). Allele calls and genotype clusters were visually inspected for errors in automated SNP genotype clustering algorithm and corrected based on the expected segregation ratio in the RIL population [48].

### RAD sequencing

High quality genomic DNA from ICCV 96029, CDC Frontier and 92 inbred lines from CPR-01 was extracted following the procedure described in Saghai-Maroof et al. [49]. Individual DNA samples were quantified using PicoGreen Assay (Life Technologies) and adjusted to 50 ng/ul. A total of 1 μg of DNA from each RIL was then used for RAD library construction following the protocol described in Baird et al. [33] and an updated method to enable pair-end Illumina sequencing (https://www.wiki.ed.ac.uk/display/RADSequencing/Home). Briefly, genomic DNA from each RIL was digested with *Eco*RI (New England Biolabs) and then ligated to a P1 adapter containing a six-bp index identifier unique to each individual. The adapter-ligated fragments were subsequently pooled, randomly sheared and size-selected between 300–500 bp on an agarose gel. The obtained DNA fragments were then ligated to a P2 adapter. To enrich RAD tags, the adapter-ligated DNA was subjected to 18 cycles of PCR enrichment followed by gel purification of the 300 to 500 bp DNA fragments. Up to 24 RAD libraries, each representing an individual RIL, were pooled for 100 bp pair-end sequencing using established v3 chemistry methodologies on single lanes of an Illumina HiSeq 2000 flow-cell (Illumina Inc.). Following sequencing and Illumina data processing (Casava v1.8.0), the valid raw pair-end reads were separated into pools using custom Perl scripts to identify reads associated with individual RILs.

Paired-end Illumina reads were demultiplexed using the FASTX toolkit's barcode splitter and PCR duplicates were removed with Samtools rmdup. Reads from each line were aligned to the chickpea genome with Bowtie [50], allowing up to two mismatches with a maximum of 600 bp between each end. SNP calling was performed using Samtools mpileup [51] allowing up to 66% missing data, a maximum of 10% heterozygosity, and allele frequency between 0.2 and 0.8. Additionally, any lines showing more than 10% residual heterozygosity were removed from further analysis. Some of the missing data was inferred by examining the allele calls flanking the missing data for a given line within a scaffold. If the flanking calls were identical we assume that no recombination occurred in that region. Based on this assumption, clusters of SNPs with identical segregation patterns were then merged and binned.

### Genetic mapping

Genotypic data generated using the RAD-seq and Illumina GoldenGate were used to create the genetic linkage map of CPR-01. Linkage analysis between the markers and the best possible linear order of the loci were determined using MadMapper [52], RECORD [53] and QTL Icimapping V3.2 (http://www.isbreeding.net/) software. Before linkage analysis, genotypic scores were filtered for missing data (genotypic score missing in more than 25% RILs and 30% per marker) and distorted allele frequency. The marker loci with allele frequencies of < 0.2 for one parent and > 0.8 for the other parent were removed from further analysis. The filtered SNP markers were clustered into linkage groups using MadMapper with recombination value (haplotype distance) cut-off of 0.2 and a BIT score of 100. Linkage groups were assigned using the position of SNP markers on the pseudochromosomes of the chickpea genome. Marker order was determined using the RECORD algorithm of RECORD_win and QTL Icimapping V3.2 software with a setting of 30 cM gap size, Kosambi mapping function and 0.1 recombination fraction allowed. Rippling was done by permutation of a window of 5 markers using COUNT rippling criteria. Following the initial map construction, double recombinants or singletons were identified as potential genotyping errors using the color genotypes feature of the MapDisto tool [54]. The potential genotyping errors and missing genotypes were inferred by using information from flanking marker data with the initial marker order. In the second round, (i.e. after error correction and infer missing genotypes) marker order was generated using the RECORD algorithm of RECORD_win and QTL Icimapping V3.2. The best marker order with the shortest linkage map distance was finally selected.

### Calculation of recombination values and genome coverage

The average genome-wide average recombination frequency in cM/Mb was calculated by dividing the total genetic map length by 323 Mb genome size flanked by the most distal markers on each linkage group. The average recombination frequency in genes/cM was calculated by dividing the total number of genes (28,269) by the map length. Recombination rates for individual chromosomes were calculated by dividing the genetic length (cM) by the sequence length (Mb) between the first and last marker placed on each chromosome. The estimated genome length was calculated using the method 4 of Chakravarti et al. [55], in which total length of the linkage groups is multiplied by the factor (m + 1)/(m-1), where m is the number of markers in the linkage groups. Furthermore according to Sekino and Hara [56] genome coverage was calculated as the ratio of total map length and estimated map length.

## Results

### Discovery and distribution of genic SNPs

In order to identify SNPs in the genic region of chickpea, eight diverse chickpea genotypes (Table 1) were selected for targeted 3’-cDNA transcript profiling using 454 Pyrosequencing technology. This process generated 4.2 million high quality (HQ) reads with an average sequence length of 472 bp (SD = 112, range = 46 to 1201). The number of raw sequence reads per genotype ranged from 496,109 reads (in cv. Amit) up to 605,001 reads (in Cr 5–10). The HQ reads were mapped against the initial draft scaffold assembly V0.1 using GMAP. Average read depth ranged from 7 to 10. In total, Samtools mpileup detected a set of 51,632 non-redundant SNPs (Table 1). Finally, 2, 279 polymorphic SNPs among the cultivated genotypes and no more than 50% missing data, were selected for SNP assay design (Additional file 1).

### Functional and structural impact of identified genic SNPs

**Figure 2:**
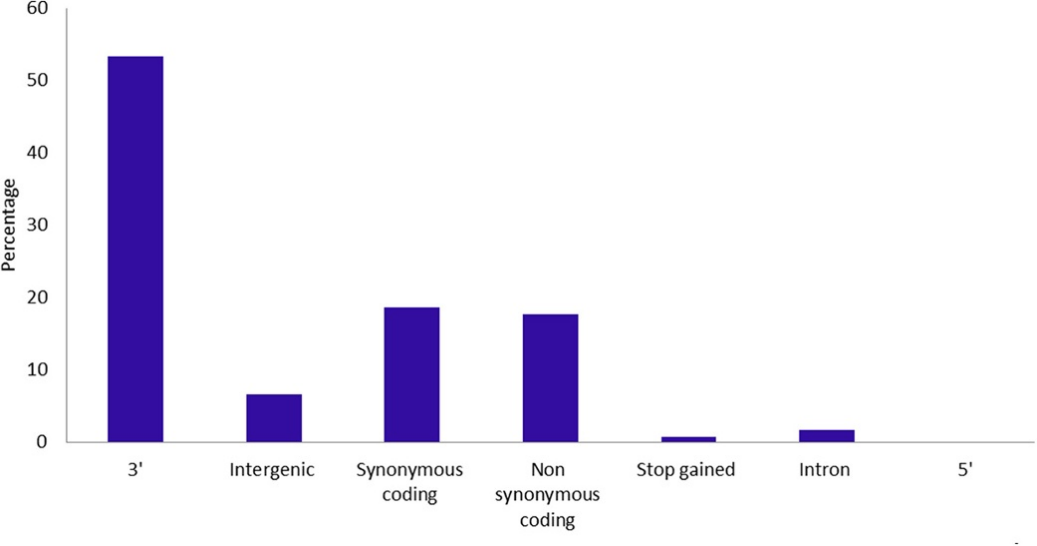
**Distribution of genic SNPs on the basis of their location in the predicted gene models of the chickpea genome.** SNPs were categorised using gene structure annotation information retrieved from the ICRISAT chickpea genome database (http://www.icrisat.org/gt-bt/ICGGC/GenomeManuscript.htm).

We investigated the functional and structural impact of the identified genic SNPs selected for assay design by comparing the position of the SNPs relative to the annotated chickpea genome. The majority (2,536 SNPs) were distributed across the eight chickpea pseudochromosomes, whereas 153 SNPs were distributed in 93 unplaced scaffolds. Only 40 SNPs could not be located in the CDC Frontier genome sequence. The average frequency of SNPs across the chickpea genome was one SNP per 138.7 Kb. The distribution of the genic SNPs was not equal for all chromosomes. The highest number of SNPs was identified on Ca4 (696) followed by Ca7 (390), Ca6 (324) and Ca1 (315), whereas the lowest number of SNPs were identified on Ca5 (137) followed by Ca8 (139), Ca3 (263) and Ca2 (272). The ratio of transitions to transversions (Ts/Tv) was 1.5, which is expected for genic regions. 53.3% of these SNPs resided downstream of an open reading frame, 37.4% in coding regions of which 18.7% were synonymous coding, 17.8% were non-synonymous coding and 0.8% generated a stop codon. Interestingly, 6.7% of SNPs resided in intergenic regions, that are located at least 5 kb up- or downstream of a gene. Further 23 SNPs in coding sequence that introduced a TAG, TAA, or TGA stop codon could potentially alter the function of these genes (Figure 2).

### Functional characterization of SNPs (gene annotation)

**Figure 3:**
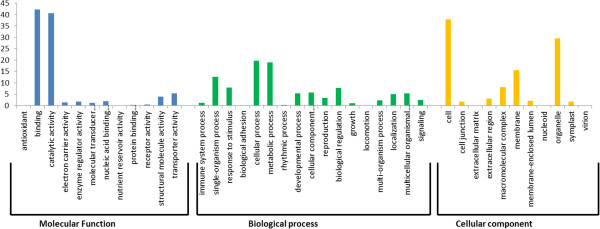
**GO annotations of SNP containing transcripts.** Annotations were grouped by three Gene Ontology classes, molecular function, biological process and cellular component. The data presented here represents the level 2 analysis and illustrating the general functional categories.

A total of 2,689 SNPs selected for assay design resided in the 1,322 genes of the CDC Frontier annotated chickpea genome. These SNPs represents an average of 1.3 SNPs per gene with a minimum of one and maximum of seven SNPs per annotated gene. 853 SNP-containing transcript sequences did not show any sequence similarity with annotated chickpea genes. SNP-containing transcript sequences were assigned GO term annotations using Blast2GO. A total of 1,056 sequences were assigned at least one GO term for describing biological processes, molecular functions and cellular components (Figure 3). For molecular function, genes involved in binding, catalytic activity and transporter activity were highly represented. Of the biological process, the major categories were cellular process, metabolic process and single-organism process. In the cellular component group, the major categories were cell, organelle and membrane.

The 1,322 SNP containing transcripts represent 464 ortholog groups (KO entries; the K numbers). The KO represents 241 pathways with maximum hits in the biosynthesis of secondary metabolites, followed by metabolic pathways. Annotation of SNP containing transcript sequences into GO and KEGG could serve as important and valuable resources for gene identification and functional analysis of some important traits in chickpea.

### Phylogenetic and diversity analysis

To investigate SNP diversity in the gene coding region that has been generated or lost during domestication and breeding, we compared SNPs between the cultivated chickpea (*Cicer arietinum*) genotype group and its wild progenitor (*Cicer reticulatum*) genotype group. A further 95 SNPs were found polymorphic between desi and wild progenitor, and 227 SNPs between kabuli and wild type chickpea. We identified 104 SNPs that were present between desi and kabuli groups.

**Figure 4:**
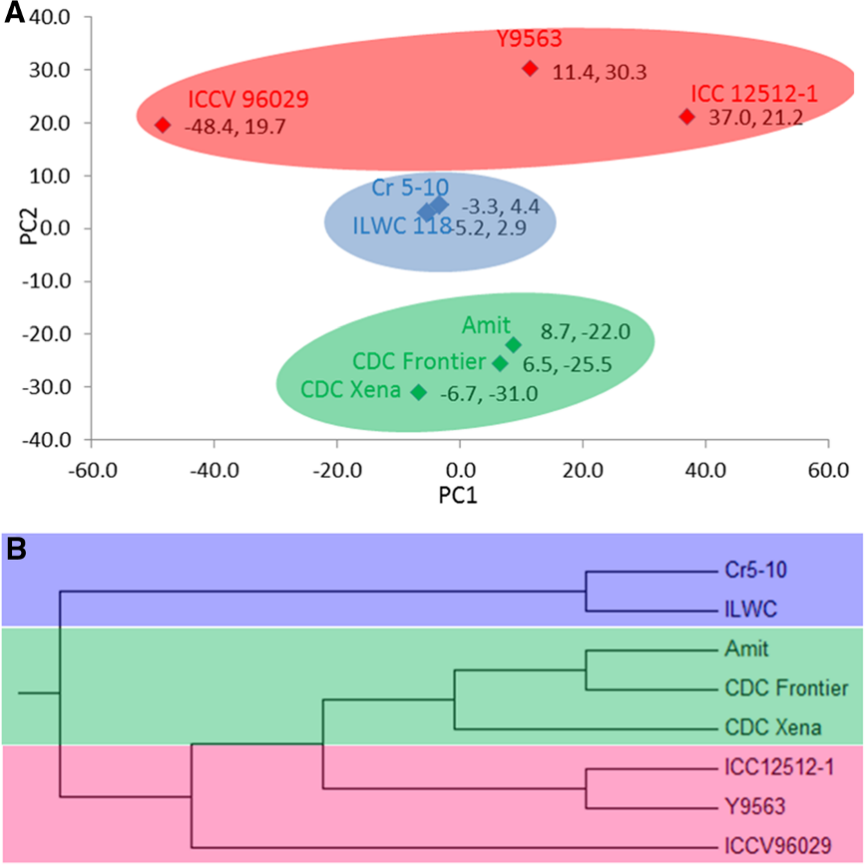
**Principal component analysis (PCA) and phylogenetic analysis using Neighbour joining (NJ) based on SNP genotypic data. A**: PCA on SNP genotypic data of the eight chickpea accessions. The X and Y-axes show PC1 (21.2%) and PC2 (20.3%), respectively. **B**: NJ tree showing genetic relationships among the chickpea accessions. The NJ-tree grouped chickpea genotypes into two major clades wild and cultivars. Further the cultivars were grouped into sub-clades, i.e., desi and kabuli types.

Population structure was analysed based on genic SNPs, using principal component analysis (PCA) and NJ-phylogenetic tree. The first three principal components explained 56.3% of the total variation and showed clustering of chickpea lines into cultivated and wild groups (Figure 4-A). The phylogenetic analysis also showed clear clades separating cultivated and wild accessions. Further, cultivated groups sub-divided into desi and kabuli type of chickpea. Desi type chickpea accession ICCV 96029 formed an intermediate clade between desi and kabuli accessions, defining its pedigree (Figure 4-B).

Microsatellites or SSRs present in the genic sequences also showed the extensive genetic diversity among the chickpea accessions. A total of 1,415 loci containing microsatellite repeats were identified. Di-nucleotide SSRs were the most abundant repeats (55.6%), followed by tri- (42.6%), tetra- (1.3%) and penta-nucleotides (0.4%). The TA and GAA repeats represented the most di- and tri-nucleotide repeats, respectively. PCR primer pairs were designed for 585 SSRs. An *in silico* analysis of SSR containing sequences showed 153 polymorphic SSRs across the eight chickpea genotypes. Di-nucleotide SSRs were more polymorphic than tri-nucleotide repeats. The number of alleles ranged from 2–4 and PIC value ranged from 0.2 to 0.6 (Additional file 2).

### Development of SNP genotyping assays and RIL genotyping

**Figure 5:**
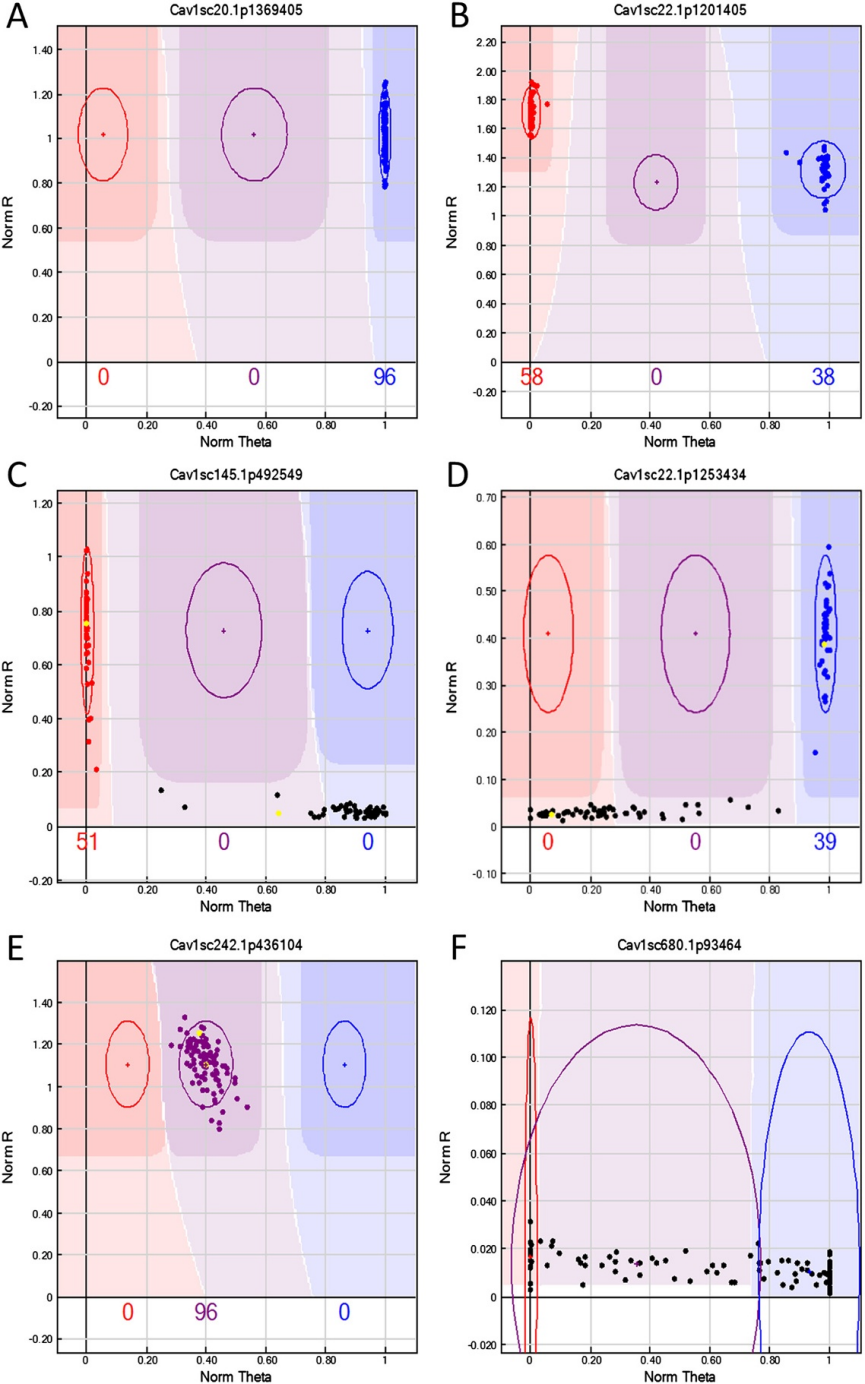
**Genotyping of CPR-01 RIL mapping population using Illumina GoldenGate genotyping platform.** Representative clustering pattern of SNP genotyping generated using the Illumina GenomeStudio software where clusters in red dots represent ICCV 96029 type allele and blue represent CDC Frontier type allele. Illustrating examples of **A**: monomorphic marker (Cav1sc20.1p369405), **B**: polymorphic marker (Cav1sc22.1p1201405), **C**: dominant type SNP (Cav1sc145.1p492549) being presence in ICCV 96029 and absence in CDC Frontier, **D**: dominant type SNP (Cav1sc22.1p1253434) being homozygous in CDC Frontier and heterozygous in ICCV 96029, **E**: heterozygote alleles for all RILs (Cav1sc242.1p436014), **F**: failed genotype pattern (Cav1sc680.1p93464). The data points in color represent genotype calls for each sample (red = AA; purple = AB; blue = BB; black = outlier) and the parents of CPR-01 population are highlighted in yellow. The x-axis (Norm Theta) represents angle of the center of cluster in normalized polar coordinate while y-axis (Norm R) represent normalized intensity.

A total of 2,729 SNPs were processed for custom OPA design with Illumina Assay Design Tool (ADT). On the basis of ADT, 2,562 (93.8%) SNPs were assigned ADT score of ≥0.6, indicating a high success rate for the conversion of a SNP into a successful GoldenGate assay. In the SNP validation process, 18 (90%) out of 20allele-specific PCR assays resulted in the identical genotype to the transcriptome sequence based SNPs (Additional file 3), thereby validating the process of SNP calling and confirming the high quality of this filtered set of SNPs. The 1,536 GoldenGate OPA was used to genotype the CPR-01 population. 1,519 SNPs (98.9%) exhibited clear interpretable clustering patterns and high GenTrain scores (mean = 0.81 ± 0.10 s.d.). Of these, 761 (49.5%) of the SNPs were polymorphic between ICCV 96029 and CDC Frontier and the remaining 750 (48.8%) were monomorphic. 17 (1.1%) SNPs failed and 8 (0.5%) had a pattern where one or another allele failed (Figure 5).

### Restriction site-associated DNA sequencing (RAD-seq)

In total, 555.6 million raw sequence reads were generated through sequencing of 92 individuals of the CPR-01 population and two parental lines in four lanes at 24-plex each lane. On average, 138.9 million raw sequence data were collected per lane; 51.4% of reads were mapped uniquely, whereas 20.9% of reads were mapped to multiple positions in the chickpea scaffold assemblies. After removing redundant reads, 22.9% of the reads were used for further analysis. A total of 233,334 raw variant SNPs across 6,556 RAD tags were identified from the collective analysis of 92 RILs against the genome assembly and provided the raw segregation data matrix for the entire population. A filtered set of 29,464 high quality SNPs (maximum heterozygosity 10%, maximum missing data 66%) were identified. After further imputation of missing data, 12,012 high-quality SNPs were selected and clustered into 626 bins with identical segregation patterns. Single SNP representing each of the 626 bins was used for linkage analysis.

**Figure 6:**
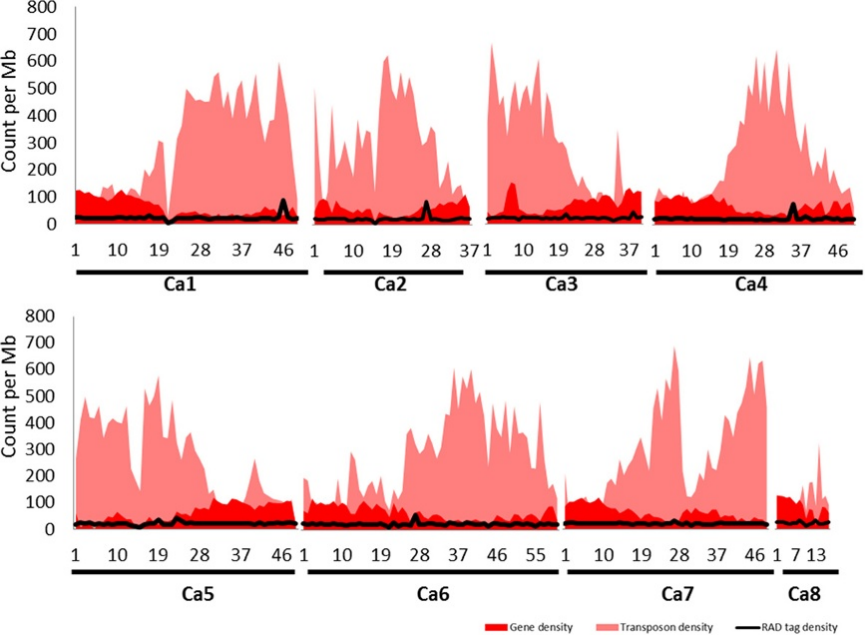
**Distribution of RAD tags across the chickpea genome.** Graph illustrates the number of RAD tags sequenced per Mb, in background with gene density and transposon density per Mb across the chickpea genome. All the transposons and genes were retrieved from the ICRISAT chickpea genome database (http://www.icrisat.org/gt-bt/ICGGC/GenomeManuscript.htm).

The number of RAD tags sequenced per Mb showed uniform genome-wide distribution across the chickpea genome (Figure 6). On average each tag was sequenced approximately 1to 235 times in every individual, indicating sufficient depth to achieve significant statistical power for SNP calling.

### High-density CPR-01 genetic linkage map

**Figure 7:**
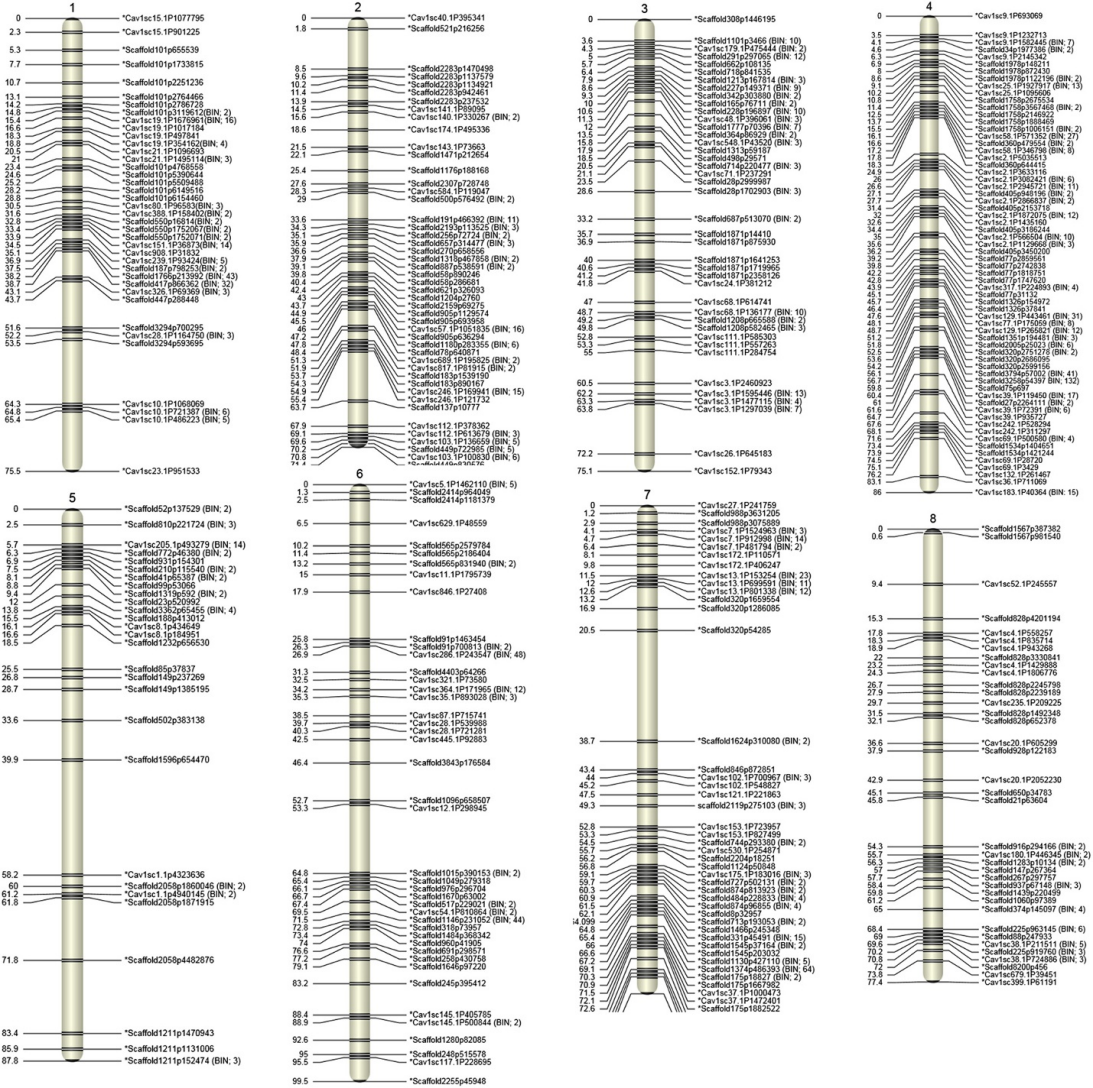
**Linkage map of chickpea based on CPR-01.** Distances of the loci (cM) are shown to the left and the names of SNP markers are shown to the right side of the linkage groups. Loci are represented as genetic BINs. The numbers of discrete polymorphic markers in corresponding BINs are noted in brackets.

A total of 92 RILs belonging to the CPR-01 population were used to construct the linkage map. A total of 1,336 SNPs out of 1,387 SNPs were mapped into eight linkage groups (Figure 7). The remaining 51 (22 RAD and 29 GoldenGate SNP) markers were not integrated into CPR-01 linkage map. CPR-01 showed a range of residual heterozygosity from 0 to 30.7%, with an average of 4.3% and median of 1.2%. Across the whole-population residual heterozygosity is 6.4%, which is 4-fold more than the expected residual heterozygosity in an F_7_-derived RIL population.

The linkage map covered a genetic distance of 653 cM, with 0.5 cM average distance between pairs of markers (Table 2). The linkage groups spanned a minimum of 71.4 cM (LG2) to a maximum of 99.5 cM (LG6). The number of loci per linkage group varied from 55 (LG5) to 429 (LG4) with a mean of 167 per linkage group. According to 322 Mb portion of the chickpea genome flanked by the most distal markers on each linkage group, the average inter-marker physical distance is 240.9 Kbp per marker. The average recombination frequency in genic regions is 43.3 genes/cM.

**Table 2:** **Summary of the CPR-01 genetic linkage map**

Linkage groups	Number of recombination BINs per LG	No of markers in BINs	Singleton markers	Total number of markers per LG	Total map distance in cM
LG1	18	149	21	170	75.5
LG2	17	87	30	117	71.4
LG3	21	112	20	132	75.1
LG4	30	394	35	429	86.0
LG5	11	38	17	55	87.8
LG6	11	124	32	156	99.5
LG7	24	195	25	220	80.2
LG8	10	30	27	57	77.4
Total	142	1129	207	1336	653.0

### Segregation distortion

**Figure 8:**
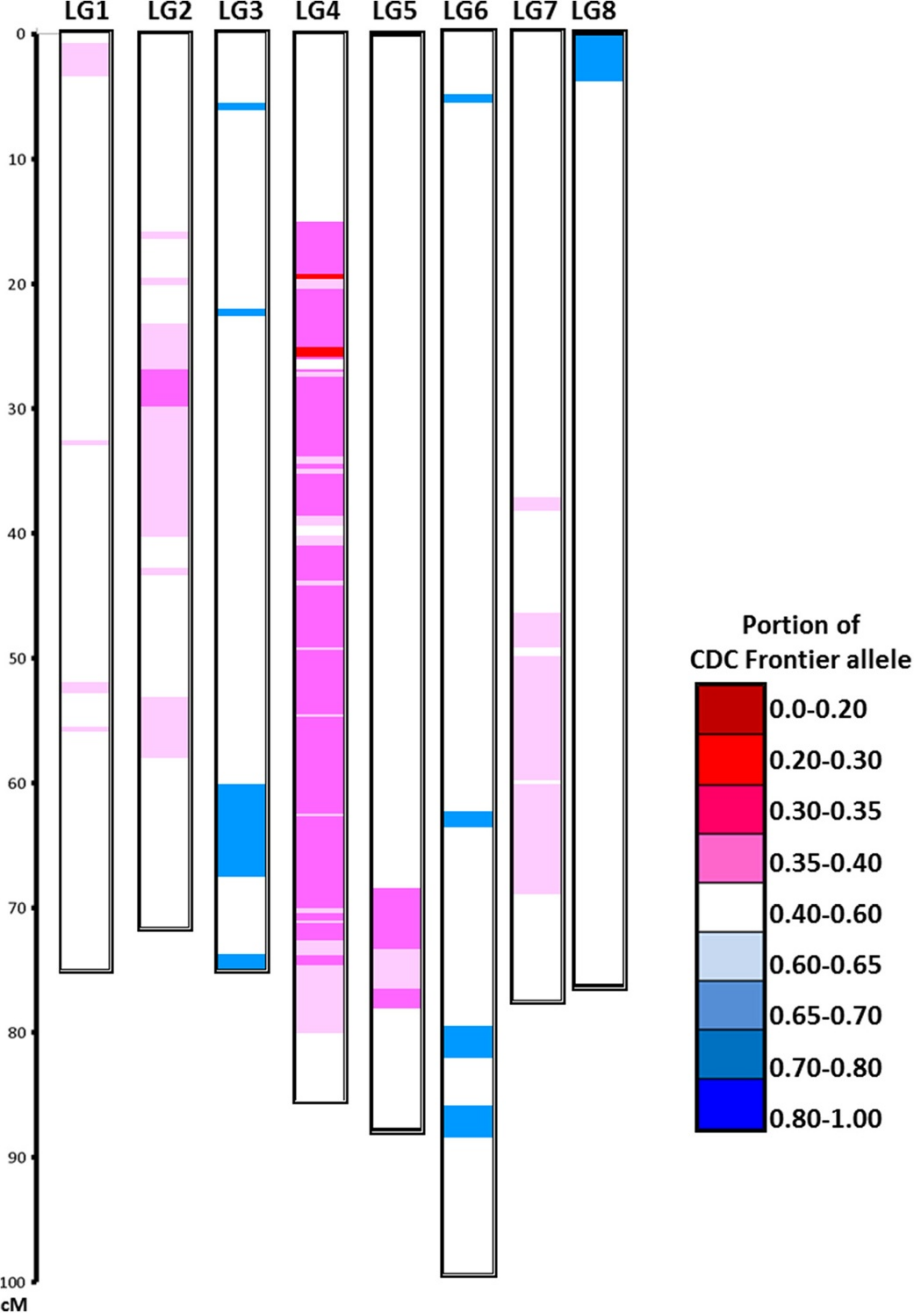
**Segregation distortion in CPR-01 population.** Genotype of 1,336 SNP loci over eight chickpea linkage groups. The proportion of CDC Frontier alleles is indicated by the color scale. White color indicates equal portion of CDF Frontier and ICCV 96029 alleles (no segregation distortion), increasing red intensity indicates significant overrepresentation of CDC Frontier alleles and increasing blue intensity indicates significant overrepresentation of ICCV 96029 alleles.

Segregation distortion was observed for 468 (35.0%) of the total mapped markers (χ2 test, p < 0.05 and allele frequency ≤0.4 and ≥0.6). Among the distorted markers, the majority (93.4%) deviated towards the female parent ICCV 96029 and 6.6% marker deviated towards the male parent CDC Frontier. This pattern of preferential transmission of ICCV 96029 alleles occurred in all linkage groups; except LG3, LG6 and LG8. LG4 showed the highest proportion of ICCV 96029 alleles (62.0% of total maternal alleles), whereas LG3 showed the highest proportion of CDC Frontier alleles (54.9% of total paternal alleles). Most of the distorted markers were clustered in specific regions on linkage groups ranging in size from 2.9 to 34.1 cM and consisting of 2 to 200 markers (Figure 8). These regions were defined as Segregation Distortion Regions (SDRs). Generally, all the markers showing segregation distortion had higher frequency towards the same parent in a given SDRs.

### Comparison of CPR-01 genetic map with chickpea pseudochromosomes

As one of the parents of CPR-01 is CDC Frontier, we compared the genetic map of CPR-01 with the CDC Frontier genome assembly. BLAST search of SNP marker sequences against the draft genome assembly assigned a total of 1,073 (80.3%) marker loci into eight pseudochromosomes and 215 (16.1%) markers into unplaced scaffolds. 12 (0.9%) markers could not be placed on the chickpea genome sequence using the BLAST search indicating that the corresponding genome sequence was missing from the published draft chickpea genome assembly. The details of mapped markers are presented in Table 3. Among the physically placed markers, 36 did not show the corresponding chromosomal assignment and are referred to as the non-syntenic markers. Some of these non-syntenic markers were singletons, but others formed clusters of 2–6 markers. For instance a cluster of 5 markers from LG4 was physically mapped to Ca6 of CDC Frontier genome. Whereas in another case, a cluster of 6 markers from LG4 was physically mapped on Ca3 of CDC frontier genome.

**Table 3:** **Physical mapping of SNP markers in the chickpea genome**

Linkage groups	Total map distance	Total number of markers	Markers mapped on unplaced scaffolds	Non-syntenic markers	Physically mapped markers on assembled chickpea genome
LG1	75.5	170	4	0	164
LG2	71.4	117	19	8	90
LG3	75.1	132	37	8	82
LG4	86.0	429	77	10	339
LG5	87.8	55	15	2	38
LG6	99.5	156	26	4	126
LG7	80.2	220	25	1	194
LG8	77.4	57	12	3	40

**Figure 9:**
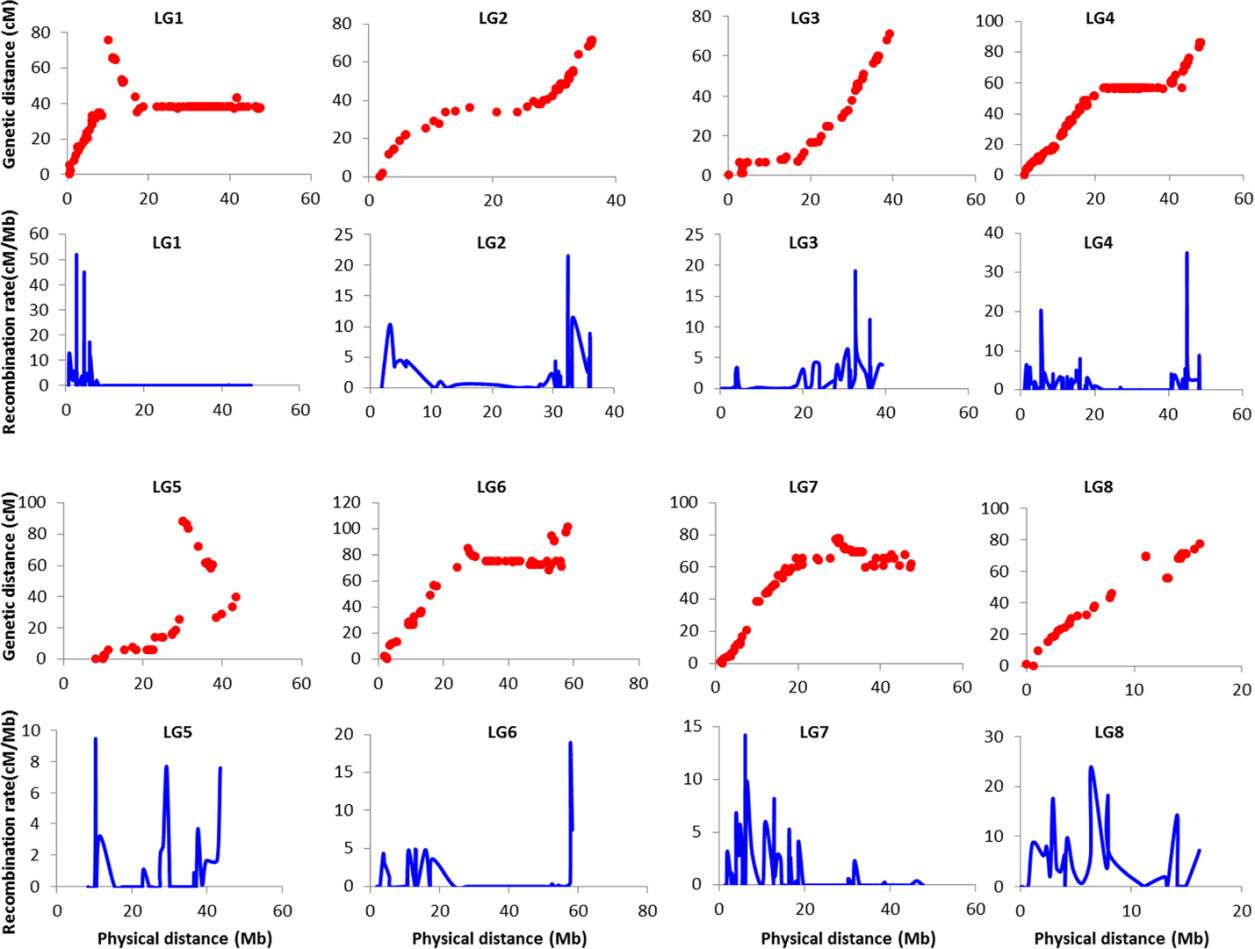
**Comparison between CPR-01 genetic map with the physical distance of eight chickpea pseudochromosomes, and corresponding recombination rates.** X-axis: physical position of the SNPs on the chickpea psudochromosome in Mb. In case of comparison between genetic and physical map Y-axis: genetic distance in cM, whereas in recombination rate maps Y-axis: recombination rate in cM/Mb.

Marker order was relatively conserved between the CPR-01 genetic map and the Cicer*_*arietinum_GA_v1.0 pseudochromosomes (Figure 9). The highest correlation between the whole genetic map and the physical map was observed for LG2 and LG3 (Spearman’s correlation coefficient 0.98), whereas the lowest correlation was observed for LG1 (Spearman’s correlation coefficient 0.69). For the rest of the linkage groups, Spearman’s correlation coefficient ranged from 0.80 to 0.97, indicating an overall good correlation between genetic and physical maps. Within some regions of the genome, inconsistency in order was observed between the genetic and physical maps. For example, in LG5, the region between 39.9-87.8 cM has negative Spearman’s correlation coefficient (−0.97) compared to the positive correlation coefficient value (0.83) of the entire LG5 indicating a possible inversion during assembly of the genome sequence. Similarly on LG8, the region between 68.4-69.6 cM has negative Spearman’s correlation coefficient (−0.85). This also points out probable errors in the chickpea sequence assembly.

### Genome wide recombination rate in chickpea

Comparison of the genetic distance based on CPR-01 linkage map and the physical distance based on CDC Frontier genome provided a snapshot of the relative range in the recombination rate along the chromosomes. The average recombination rate across the chickpea genome was 2.0 cM/Mb (Table 4). Recombination rates for individual chromosomes ranged from 1.6 to 4.8 cM/Mb. LG8 has the maximum recombination rate of 4.8 cM/Mb and LG1 the lowest at 1.6 cM/Mb likely due to the large inversion occurred on the LG1. In contrast to the average recombination rate of the entire chromosome, the recombination rate within chromosomes varied considerably, with a range of 0 to 52 cM/Mb. Chromosomal regions with a high recombination rate (>20 cM/Mb) were considered as ‘hot spots’ for recombination. At least five hot spots were detected on chromosomes Ca1, Ca2, Ca4 and Ca8. There were also several regions with moderately high (10–19 cM/Mb) recombination rate. The highest recombination rate was observed in an interval between scaffold101p2764466 and scaffold101p2786728 on Ca1, showing a genetic distance of 2.5 cM and physical distance of 22.2 Kb. As the centromeres and their flanking pericentromeric regions are not precisely defined in the chickpea genome, the regions with 0 cM/Mb where recombination was completely suppressed can be considered ‘cold spots’ (Table 5). In order to avoid conflict between centromeric regions and recombination cold spots, we have not analysed the cold spots in detail in this research. However, one of the chromosomal regions in Ca6 was used to compare the region with high and low recombination rates. This chromosomal region includes a recombination ‘cold spot’ (0 cM) that extends from scaffold1146p231052 to scaffold130p30393, with a corresponding physical distance of 22.8 Mb. This ‘cold spot’ has a low gene density of 39.6 genes per Mb as compared with gene density of 66.2 genes per Mb across the whole chickpea genome.

**Table 4:** **Summary of the recombination rate on individual LGs and across the chickpea genome**

Linkage group	Map distance(cM)	Distance between first and last marker in LG (Mb)	Recombination rate (cM/Mb)
LG1	75.5	46.9	1.6
LG2	71.4	34.3	2.1
LG3	75.1	39.1	1.9
LG4	86.0	47.5	1.8
LG5	87.8	35.3	2.5
LG6	99.5	56.5	1.8
LG7	80.2	46.3	1.7
LG8	77.4	16.1	4.8
Total	652.9	321.9	2.0

**Table 5:** **Recombination hotspots and cold spot and their genomic composition**

Chr.	Flanking markers	Interval in cM	Interval in bp	Recombination rate (cM/Mb)	G + C content (%)	Number of genes	Gene density per Mb
**Recombination Hot spots**							
Ca1	scaffold101p2764466-scaffold101p2786728	13.1-14.3	22262	53.9	30.2	2	89.8
Ca2	CAV1SC817.1P81915-scaffold183p1539190	51.9-53.7	84368	21.3	27.5	8	94.8
Ca4	CAV1SC25.1P1095606-scaffold1758p2675534	10.2-10.8	27999	21.4	31.5	6	214.3
Ca4	scaffold1534p1404651-scaffold1534p1421244	73.4-73.9	16593	30.1	29.7	2	120.5
Ca4	CAV1SC69.1P28720 -CAV1SC69.1P3429	74.5-75.1	25188	23.8	26.8	2	79.4
Ca8	CAV1SC20.1P605299-scaffold928p122183	36.6-37.9	55103	23.6	27.6	6	108.9
**Recombination Cold spot**							
Ca6	scaffold1441p53604-scaffold213p601853	75.1-75.1	22,875,656	0	22.1	906	39.6

## Discussion

### SNP discovery and genotyping

Gene based molecular markers have increasingly been used in genetic mapping and in breeding programmes for marker-assisted selection (MAS) in several crops. Over the last few years NGS platforms have accelerated the process of large scale SNP discovery see review by [57] leading to the development of large numbers of SNP-based markers. Here we identified 2,979 high quality SNPs in cultivated (desi and kabuli type) and wild chickpea genotypes using *Aci* I digested 3’-anchored cDNA profiled by 454 sequencing. 3’-anchored cDNA profiling, where cDNA is sequenced from the extreme 3’ end of transcript, provides an accurate analysis of mRNA distribution due to the high read-depth representation [27, 40]. The larger representation of 3’ UTR-located SNPs compared to coding region SNPs (53.3% vs. 37.0% respectively), combined with the higher read-depth, leads to the higher prospect of identifying SNPs using 3’-anchored cDNA sequencing compared to sequencing of randomly sheared cDNA fragments.

One of the important reasons behind the utilization of genic SNP markers in genetic mapping is its potential to establish a direct link between important agronomical traits to the functional SNPs and also to find candidate genes underlying traits of interest. Several genic SNPs markers with significant association with traits have been identified in many plant species [58, 59]. In chickpea, SNPs from five different candidate genes (*ERECTA*, *ASR*, *DREB*, *CAP2* and *AMDH*) were found significantly associated with morphological, phenological**,** yield and yield related traits [60]. Recently Thompson and Tar’an identified a SNP within the *acetohydroxyacid synthase* 1(*AHAS1*) gene which confers resistance to imidazolinone (IMI) herbicide and also developed a breeder friendly allele-specific SNP marker for use in marker-assisted selection (MAS) for IMI-resistant in chickpea [61]. These findings suggest that genic SNPs can be used as functional markers to establish the link with traits and can be potentially used in marker assisted selection of genotypes with the desired alleles.

We used three genotypes of each desi and kabuli market class and two wild chickpea accessions for genic SNP discovery. The average allele frequency of the identified SNPs was 4.9% for kabuli accessions and 8.8% for desi accessions. This difference reveals that desi accessions used in this study were more genetically diverse then the kabuli accessions. In contrast Upadhyaya et al. [62] observed more genetic diversity in kabuli accessions than in desi accessions using 48 SSR markers. The limited sample size used in the current study makes it difficult to draw any conclusion about SNP prevalence in either type of cultivated chickpea. The 8 accessions used in the analysis were selected as the most diverse materials (based on pedigree, phenotype data and some initial marker analysis) from a pool of breeding lines and germplasm collection available to us. We believed that these lines although relatively small in number may represent the true diversity in the larger pool of genotypes. The identified genic SNPs were able to determine genetic relationships among the selected diverse chickpea accessions. The phylogenetic analysis based on SNP diversity identified clear clustering of chickpea accessions based on species type. Cultivated and wild type accessions were clearly separated. Within cultivated accessions desi type and kabuli type also form separate clusters, whereas ICCV 96029 was placed intermediate between the desi and kabuli clusters. The pedigree of ICCV 96029 justifies the phylogenetic relationship of ICCV 96029 with both desi and kabuli accessions as ICCV 96029 is derived from a complex cross between five different desi (K-850, Gw-5/7 and XP-458) and kabuli accessions (L-550 and Guamuchil). Overall, these results are in general congruence with the earlier genetic diversity studies [63] and demonstrate the utility of identified genic SNPs for studying genetic diversity in chickpea.

The effectiveness and suitability of the Illumina GoldenGate SNP genotyping platform has been well demonstrated in several crops, including chickpea [11]. Our study provides an additional set of 1,536 SNPs that can be used for genotyping mapping populations and other genetic resources of chickpea. The SNP genotyping success rate was high, with 98.9% SNPs showing clear scorable clusters. The higher genotyping success rate than previously reported in chickpea (90.8%) [11], pea (91.0%) [64], and lentil (84.0%) [27] is attributed to the SNP calling criteria and SNP selection process employed in the assay design in this study.

Genetic mapping with gene-based SNP markers in chickpea genome consist of >45% repetitive elements of the total nuclear genome may result in uneven coverage of the genetic map. Therefore, in addition to genic SNP based GoldenGate genotyping arrays, we used RAD-seq to construct a high-density genetic linkage map. For the precise anchoring of the newly sequenced genome assembly, a high genome-wide marker density is necessary. The RAD-seq methodology utilized in this study has advantages over other GBS methods in this regard. The use of *Eco*RI for RAD-Seq yields a uniform distribution of sequence reads across the genome that enables a greater coverage of the assembly. In contrast, GBS methodologies commonly utilized methylation-sensitive restriction enzymes that cannot cleave the repetitive regions of the genome. This provides not only a targeted coverage of the gene rich regions of the genome but also potentially missed portions of the assembly. A comparison of the two methodologies for the purposes of anchoring a genome assembly has been provided recently for *Brassica oleracea* [65].

### High-density genetic map using array-based genic SNPs and RAD tags

This study generated one of the most comprehensive intraspecific linkage maps of chickpea to date. The map spans 653 cM and is divided into eight linkage groups corresponding to the number of chickpea chromosomes, with average inter-marker distance of 0.5 cM. In comparison, earlier intraspecific linkage maps [12, 66] were sparsely covered with 230 and 408 markers, spanning 740 cM and 752 cM with average inter-marker distance of 3.2 and 2.16 cM, respectively. Several interspecific linkage maps have been developed using second generation sequencing technologies e.g., [11, 21]. The most saturated chickpea reference genetic linkage map derived from a interspecific cross between ICC 4958 (*Cicer arietinum*) and PI 489777 (*Cicer reticulatum*), thus far is comprised of 1,291 markers, spanning 846 cM with an average inter-marker distance of 0.65 cM [20]. The intraspecific CPR-01 linkage map (Figure 6) covers more than 99% of the chickpea genome and has an inter-marker distance of 0.5 cM, indicating the immense potential of this sequence-based mapping strategy for anchoring and detecting or correcting the orientation of scaffolds to the chromosome. The high density genetic maps can also greatly improve the precision of QTL mapping.

Segregation distortion is a common phenomenon in mapping populations which the frequency of genotypes skews from the expected Mendelian ratio [67]. In the CPR-01 mapping population, 468 (35.0%) of the mapped markers showed segregation distortion and the markers were retained in the map. Most of these distorted loci corresponded to regions already reported as prone to segregation distortion in previous studies [66, 68–70]. In CPR-01, the majority of markers 325 (75%) located on LG4 showed distorted segregation toward the maternal parent ICCV 96029. Earlier reported studies using F_2_ and RIL mapping populations also observed several distortion regions on LG4 [71]. However, in this study with a high-density linkage map we were able to plot the location of SDLs with a high degree of precision. LG4 contains several QTLs for important agronomical traits [9, 72, 73].

### Genome wide recombination rate in chickpea

The recombination rate varies by an order of magnitude among species and between individuals [74, 75]. Several studies have been conducted to attempt to understand the factors involved in genetic recombination rates and sequence features that may correlate with this variability. In many eukaryotic species, a positive correlation between GC content and recombination rate was observed [74]. It was also observed that, the gene rich regions of wheat, barley, and maize, are more recombinationally active than gene-poor regions [76, 77]. However, in the current study we found no relationship between recombination frequency and GC content (Table 5). The average GC content of all identified recombination hot spots (29.2%) was similar to the overall GC content of the chickpea genome (30.8%). Though, all the identified recombination hot spots showed higher gene density than the recombination cold spots and the gene density across the genome. Similar observations were also reported in rice and wheat [78, 79]. On the other hand, a weak negative or lack of correlation between gene density and recombination rates has also been reported in Arabidopsis [77, 80]. The comparison between genetic and physical distances has provided initial ‘landscape’ information about the recombination rate and variation in the CPR-01 population. The recombination rates calculated for CPR-01 do not necessarily apply to the other chickpea population as the recombination rate varies substantially between different crosses and could reveal different general and location-specific levels of recombination [30]. However, understanding the detailed landscape of recombination could provide information for marker-assisted selection strategies for specific traits in chickpea. Further experiments with analyses in different genetic backgrounds are needed to confirm the strength and the precise location of these hot spots.

### Genome wide comparison between genetic map and the chickpea genome sequence

Alignment of the CPR-01 map with the chickpea pseudochromosomes indicated overall high co-linearity between the genetic and the physical order. However, the alignment also showed some inconsistency in localized order on Ca1, Ca5, Ca6, Ca7 and Ca8. This could be due to the incorporation of small scaffolds and/or too little recombination to allow exact placement or correct orientations of scaffolds during the chickpea genome assembly. Another possible reason of marker order inconsistency could be due to the utilization of an interspecific genetic map generated by using a cross between *Cicer arietinum* X *Cicer reticulatum* for the genetic anchoring of the chickpea pseodochromosomes (Cicer_arietinum_GA_v1.0). In spite of substantial morphological similarities and crossability between C*. arietinum* and *C. reticulatum*, some chromosomal rearrangements such as reciprocal translocation, a paracentric inversion or location of chromosomal satellites have been reported [81]. Also, comparative mapping of *C. arietinum* X *C. reticulatum* genetic map using common SSR markers also detected a few inversions in marker order possibly due to inversion of DNA sequences and minor chromosomal translocation [66, 82]. Therefore the observed marker order inconsistency in our analysis could potentially represent the reported chromosomal rearrangement between *C. arietinum* and *C. reticulatum*. Further analysis is needed to test this hypothesis.

NGS has dramatically increased the rate of the completion of new genome sequences across different species. Most of the recently released plant genomes were sequenced using NGS platforms see “The First 50 Plant Genomes” review by [83]. Sequencing and genome assembly using NGS is a challenging task, especially for large eukaryotic genomes [84, 85]. In order to improve these reference genome sequences, they need to undergo quality improvement to repair the assembly errors, as has been undertaken in the maize, Brassica and Arabidopsis genomes. With the availability of low cost genotyping technologies, many high density alternative reference genetic maps have been generated and detailed comparisons of genetic maps and genome sequences have been conducted. These multiple reference genetic maps have all revealed some degree of physical assembly error and missing fragments in the reference genomes [24, 86]. With the high density chickpea genetic map generated in this study, it is now possible to locate genomic regions which less accurately placed using the earlier genetic map used in the chickpea genome assembly. Similarly, this high density map allows independent checking and validation of the current chickpea genome assembly. For example some of the major inversions on Ca5 and Ca8 can potentially be corrected using the current high density CPR-01 genetic map.

## Conclusion

This study generated a high-density intraspecific linkage map of chickpea using genic SNP based genotyping assay and RAD-seq GBS. The map allowed addressing some issues with marker alignment in the corresponding chromosome and inconsistency in marker order within the physical map. The high-density CPR-01 map helped in assigning large number of previously unplaced scaffolds from the version 1.0 of the CDC Frontier draft genome sequence. The alignment analysis also revealed the varying degrees of recombination rates and hotspots across the chickpea genome. On average the estimated genome-wide recombination rate in the current population is 2.0 cM/Mb ranging from 1.6 to 4.8 cM/Mb per chromosome.

The CPR-01 is one of the key genetic materials derived from an intraspecific cross segregating for some important agronomic traits in chickpea, such as photoperiod sensitivity, ascochyta blight resistance and double podding. Therefore the high-density CPR-01 map will help to precisely map and estimate the effects of quantitative trait loci for these traits. Furthermore, the information on the genome wide recombination rates in CPR-01 may provide the basis for designing effective marker-assisted selection strategies.

### Availability of supporting data

The data sets supporting the results of this article are included within the article as additional files.

### Data availability

Sequence data under the study has been deposited into NCBI Sequence Read Archive (http://www.ncbi.nlm.nih.gov/sra) accessions SRP044343 and SRP044316.

## Electronic supplementary material

Additional file 1: **SNP data including list of high quality SNPs, reference base, alternate base, allele frequency and flanking 60 bp nucleotide sequences.** (XLS 1 MB)

Additional file 2: **List of Identified SSR, providing details on SSR motifs, primer sequences, expected PCR product size and** ***in silico*** **Polymorphism survey in eight chickpea genotypes.** (XLSX 1 MB)

Additional file 3: **List of SNPs selected for KASP assays, and the results of these assays on a panel of 8 chickpea lines.** (XLSX 16 KB)
